# Development of a Single-Chain Variable Fragment of CR3022 for a Plasmonic-Based Biosensor Targeting the SARS-CoV-2 Spike Protein

**DOI:** 10.3390/bios12121133

**Published:** 2022-12-06

**Authors:** Taufik Ramdani Tohari, Isa Anshori, Umi Baroroh, Antonius Eko Nugroho, Gilang Gumilar, Shinta Kusumawardani, Sari Syahruni, Brian Yuliarto, Wyanda Arnafia, Irvan Faizal, Yeni Wahyuni Hartati, Toto Subroto, Muhammad Yusuf

**Affiliations:** 1 Research Center for Molecular Biotechnology and Bioinformatics, Universitas Padjadjaran, Bandung 40133, Indonesia; 2 Lab-on-Chip Group, Biomedical Engineering Department, Institute of Technology, Bandung 40132, Indonesia; 3 Research Center for Nanoscience and Nanotechnology (RCNN), Institut Teknologi Bandung, Bandung 40132, Indonesia; 4 Department of Biotechnology, Indonesian School of Pharmacy, Bandung 40266, Indonesia; 5 Advanced Functional Material Research Group, Faculty of Industrial Technology, Institut Teknologi Bandung, Bandung 40132, Indonesia; 6 Research and Development Division, PT. Biostark Analitika Inovasi, Bandung 40375, Indonesia; 7 Research and Development Division, PT. Tekad Mandiri Citra, Bandung 40292, Indonesia; 8 Centre for Vaccine and Drug Research, National Research and Innovation Agency Republic of Indonesia, Kawasan Puspiptek Serpong, Tangerang Selatan 15314, Indonesia; 9 Department of Biotechnology, Faculty of Biotechnology, Atma Jaya Catholic University of Indonesia, BSD Campus, Tangerang 15345, Indonesia; 10 Department of Chemistry, Faculty of Mathematics and Natural Sciences, Universitas Padjadjaran, Jatinangor 45363, Indonesia

**Keywords:** molecular dynamics, portable SPR, plasmonic-based bioreceptor, SARS-CoV-2, scFv

## Abstract

Two years after SARS-CoV-2 caused the first case of COVID-19, we are now in the “new normal” period, where people’s activity has bounced back, followed by the easing of travel policy restrictions. The lesson learned is that the wide availability of accurate and rapid testing procedures is crucial to overcome possible outbreaks in the future. Therefore, many laboratories worldwide have been racing to develop a new point-of-care diagnostic test. To aid continuous innovation, we developed a plasmonic-based biosensor designed explicitly for portable Surface Plasmon Resonance (SPR). In this study, we designed a single chain variable fragment (scFv) from the CR3022 antibody with a particular linker that inserted a cysteine residue at the second position. It caused the linker to have a strong affinity to the gold surface through thiol-coupling and possibly become a ready-to-use bioreceptor toward a portable SPR gold chip without purification steps. The theoretical affinity of this scFv on spike protein was −64.7 kcal/mol, computed using the Molecular Mechanics Generalized Born Surface Area (MM/GBSA) method from the 100 ns molecular dynamics trajectory. Furthermore, the scFv was produced in *Escherichia coli* BL21 (DE3) as a soluble protein. The binding activity toward Spike Receptor Binding Domain (RBD) SARS-CoV-2 was confirmed with a spot-test, and the experimental binding free energy of −10.82 kcal/mol was determined using portable SPR spectroscopy. We hope this study will be useful in designing specific and low-cost bioreceptors, particularly early in an outbreak when the information on antibody capture is still limited.

## 1. Introduction

Timely diagnosis, effective treatment, and future prevention are keys to managing future outbreaks, as a lesson learned from the COVID-19 pandemic. The current race to develop cost-effective contact-point diagnostic tests and efficient laboratory techniques to confirm SARS-CoV-2 infection has set a new frontier in diagnostic innovation. The popular commercial COVID-19 diagnostic tests fall into two major categories. The first category is qRT-PCR, quantitative reverse transcription-polymerase chain reaction, a nucleotide-based test of viral RNA [1,2]. The second category is rapid antigen tests, which are fast and easy to perform and can serve for high-throughput routine screening or triage of infected individuals. These antigen tests are suitable up to five to ten days after symptom onset while viral loads are high, indicating the virus’s presence, not residual RNA [3,4,5].

To aid continuous innovation, we developed a plasmonic-based biosensor designed explicitly for portable SPRs. This biosensor can improve COVID-19 diagnosis by offering higher sensitivity and specificity in shorter response times, with real-time monitoring to uncover binding dynamics. Moreover, it allows label-free measurement of analytes. In addition, the portable SPR (nano-SPR8, NanoSPR LLC, USA) device is a simpler version of the SPR instrument that is easy to carry due to its small size, potentially providing point-of-care testing (POCT) [6,7,8,9,10,11].

There are major aspects in developing a biosensor for diagnostic tools. Typically, a bioreceptor is a recognition element that selectively captures analytes. A transduction element, generally a group of transition metals, e.g., gold, converts recognition into a measurable signal [9,12]. In the case of SARS-CoV-2, the spike (S) protein is the key target of neutralizing antibodies located on the virus’s surface and plays a critical role in viral infection [13,14,15]. Therefore, S1, the S protein subunit containing the immunologically receptor-binding domain (RBD), became the target for detection of SARS-CoV-2 [16,17].

The RBD of SARS-CoV-2 as a biomarker was recognized by scFv, an antibody-derived single-chain fragment variable. Engineered recombinant antibody fragments are increasingly used in medical diagnosis and therapy for many diseases. The advantages of scFv are its small size, low immunogenicity, high specificity, and ability to be genetically modified. Although scFv is smaller than full-length IgG, it retains a complete antigen-binding site. Due to its small size, scFv is easily produced in bacterial expression systems. Several scFvs have been established to control viral infections, including scFvs against chicken infectious bursal disease virus, scFvs targeting the H5N1 human influenza virus, and scFvs against phosphoproteins from Newcastle disease virus. Thus scFv is considered a potential bioreceptor for detecting viral diseases [10,11,18].

In this study, we aimed to develop an scFv from the CR3022 antibody to be used in a plasmonic-based biosensor using protein modeling and molecular dynamics simulation and to express it in *Escherichia coli* BL21 (DE3). Furthermore, the activity of scFv was evaluated using spot tests and portable SPR spectroscopy. Our results provide a basis for the development of an scFv-plasmonic-based biosensor to capture protein S of SARS-CoV-2, which could be useful for other protein targets as well.

## 2. Materials and Methods

### 2.1. Materials

Materials used in this study include synthetic genes (Novagen, Madison, WI, USA); Bovine Serum Albumin, BSA (Sigma Aldrich St. Louis, MO, USA); Isopropyl β-d-1-thiogalactopyranoside, IPTG (Sigma Aldrich); Sucrose (Sigma Aldrich); Ethylenediaminetetraacetic acid, EDTA (Sigma Aldrich); Terrific Broth, TB (Himedia, Kennett Square, PA, USA); Tris-base (Promega, Madison, WI, USA); Tween-20 detergents (Sigma Aldrich); Membrane Strip Test (Cytiva, Marlborough, MA, USA), Protein Marker (Bio-Rad Precision Plus Protein™ Dual Color Standards); Inactivated Transfer Media, ITM (NEST Scientific, Woodbridge, NJ, USA); Phosphate-Buffered Saline Tablets, PBS (Sigma Aldrich); Bare gold chip (NanoSPR LLC, Chicago, IL, USA); Spike Receptor Binding Domain SARS-CoV-2, RBD (Invitrogen, Waltham, MA, USA); and ethanol (Merck, Darmstadt, Germany).

### 2.2. Methods

#### 2.2.1. Molecular Design of scFv

1. Structure Modeling of scFv

**Figure 1:**
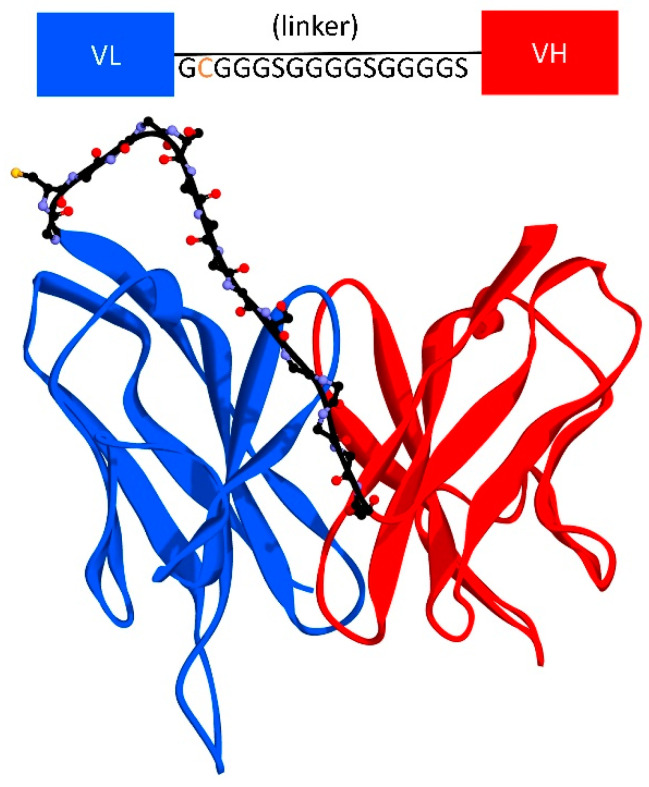
Molecular design of scFv.

The scFv CR3022 was constructed from a crystal structure of CR3022 Fab-RBD of SARS-CoV-2 (PDB: 6W41). The VL and VH domain of CR3022 were extracted from the Fab structure as an Fv fragment and connected by a peptide linker containing cysteine (Figure 1). Furthermore, the complete structure of scFv CR3022 was modeled using MODELLER 9.19 with PDB 6W41 as a template [19]. The quality of this model was evaluated with a Ramachandran plot using the PROCHECK server, VERIFY-3D method, and Z-Score using ProSA-web [20].

2. Molecular Dynamics Simulation

The binding affinity of scFv CR3022-RBD SARS-CoV-2 was evaluated by molecular dynamics (MD) simulation. The system was prepared using pdb4amber in AMBER18 [21], including the cysteine and histidine types. There were two disulfide bridges in scFv and four disulfide bridges in RBD SARS-CoV-2. The TIP3P box water molecule model was added to the system along with chloride for ion neutralization. The first minimization was performed using the steepest descent algorithm for 1000 steps. Next, 2000 steps of the conjugated gradient with 500 kcal/molÅ^2^ of harmonic restraint were applied for the backbone atoms. The unrestrained conjugate gradient then performed a final 1000-step minimization. The system was gradually heated to reach 298 K (25 °C) with NVT ensemble using a harmonic restraint of 5 kcal/molÅ^2^ on the backbone atoms. Then, the restraint was gradually released by 1 kcal/molÅ^2^ until it reached 0 for 1000 ps of NPT equilibration. Finally, the production stage was run for 100 ns with the time step of 2 fs in an NPT (Barendsen barostat) ensemble with all hydrogen atoms constrained using the SHAKE algorithm. The nonbonded cut-off value was used at 9 Å, and the long-range electrostatics was treated using the Particle Mesh Ewald. The MD trajectories were analyzed using the CPPTRAJ module in AmberTools. The binding energy calculation was performed using the MM/GBSA method with 1 ns of interval step and 150 mM of implicit salt concentration.

#### 2.2.2. Production of scFv

The synthetic genes were inserted into a pET22b(+) vector containing a pelB promoter for controlling periplasmic protein expression (Novagen, Madison, WI). For scFv expression, *E. coli* BL21 (DE3) cells were transformed with recombinant plasmids. The transformed *E. coli* BL21 (DE3)/pET-20b(+)-scFv cells were cultured at 28 °C in Terrific Broth (TB) medium supplemented with 100 μg/mL ampicillin until OD600 reached 1.0. Then, 0.5 mM Isopropyl β-d-1-thiogalactopyranoside (IPTG) was introduced at 28 °C for 4 h. Soluble scFv was extracted from *E. coli* periplasm using Tris/Sukrosa/EDTA buffer (TSE buffer). The scFv was characterized using 12% polyacrylamide gel electrophoresis.

#### 2.2.3. Spot-Test Analysis

Spot-test analysis was used to determine the binding activity of scFv CR3022 to RBD SARS-CoV-2 compared to other proteins. The binding activity of scFv was examined by immobilizing 0.5 mg/mL of the scFv on the membrane strip, periplasmic protein *E. coli* BL21 (DE3) expressed natively, and 0.5 mg/mL BSA as a negative control. Furthermore, the RBD conjugates were spotted on the conjugate pad, part of the membrane strip. For the test, 50 µL running buffer (50 mM Tris buffer containing 0.05% Tween-20 pH 7.5) was applied to the membrane strip.

#### 2.2.4. Binding Analysis Using Portable Surface Plasmon Resonance (SPR)

SPR spectroscopy was used to determine the binding affinity of scFv CR3022 to RBD SARS-CoV-2 and the success or failure of the scFv immobilization process on the SPR gold chip. The SPR experiments were performed with a nanoSPR8 instrument from NanoSPR LLC, USA. The bare gold chip was cleaned by immersing it in absolute ethanol, followed by deionized water, and then dried. The scFv was immobilized on the sensor surface by a thiol-based ligand, then incubated for 90 min and washed with PBS (1x, pH 7.2). The blocking solution and BSA (1% *w/v*) were immobilized on the sensor surface, incubated for 10 min, and washed with PBS.

For baseline SPR measurement, the running buffer of PBS was mixed with Inactivated Transfer Media and was allowed to flow over the sensor surface for 6 min. For the association and dissociation measurement, various concentrations of RBD that were arranged at 50, 100, 150, and 200 ng/mL were allowed to flow over the sensor surface for 9 min. Afterward, the concentrations flowed back for 9 min. All of the flow cells were set at 20 µL/min. Finally, the sensorgram was analyzed with the adsorption isotherm models using non-linear regression to obtain the affinity-binding parameter.

## 3. Results

### 3.1. Molecular Design of scFv

The 16-amino-acid linker connected the C-terminus of VH to the N-terminus VL by a G(C)(Gly4Ser)3 linker (Figure 1). In addition, a cysteine residue was inserted at the second position of the linker.

**Figure 2:**
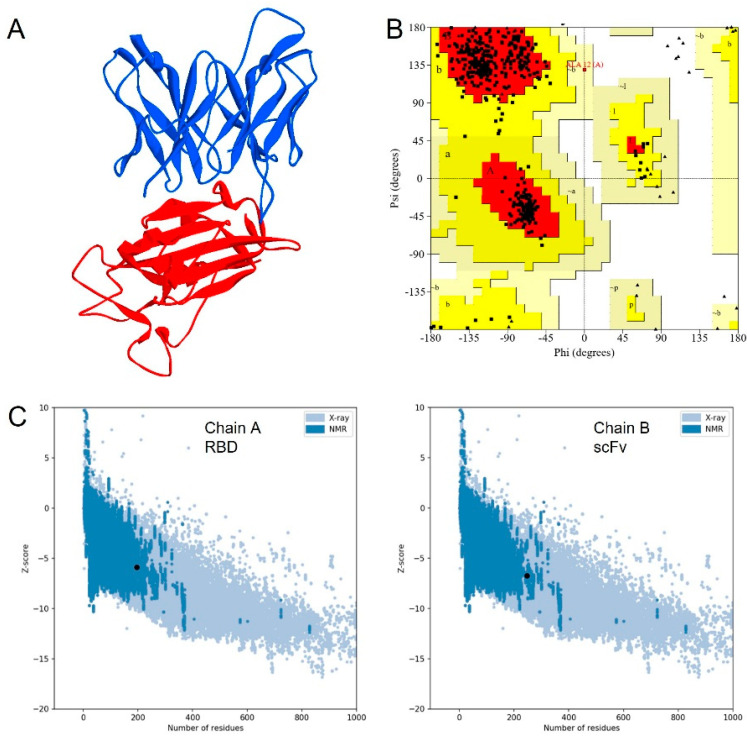
Structure model of scFv in blue and RBD of SARS-CoV-2 in red (**A**); Model assessed by Ramachandran plot (**B**) and z-score analysis (**C**).

The scFv model was constructed using an existing structure complex of CR3022 against the RBD of SARS-CoV-2 in PDB code 6W41 at 3.1 Å resolution as a template (Figure 2A). The quality of the model was assessed by Ramachandran plot, VERIFY-3D, and Z-score. The Ramachandran plot showed that 89.1% of residues were located in the most favored regions, whereas 10.7% and 0.3% residues were in the additionally allowed and generously allowed regions, respectively (Figure 2B). None of the residues were located in the disallowed region. From the VERIFY-3D analysis, 94.34% of residues had an averaged 3D-1D score of more than 0.2, indicating an acceptable model. The Z-score analysis was also located in the range of NMR or X-ray structure (Figure 2C), which shows that the model has good quality [20].

### 3.2. Molecular Dynamics Simulation

**Figure 3:**
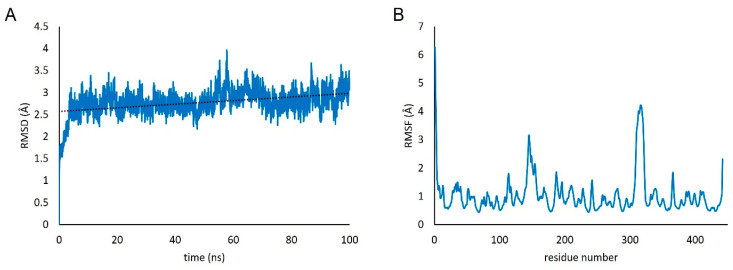
RMSD of protein backbone (**A**) Residual RMSF profile throughout 100 ns of simulation (**B**).

**Figure 4:**
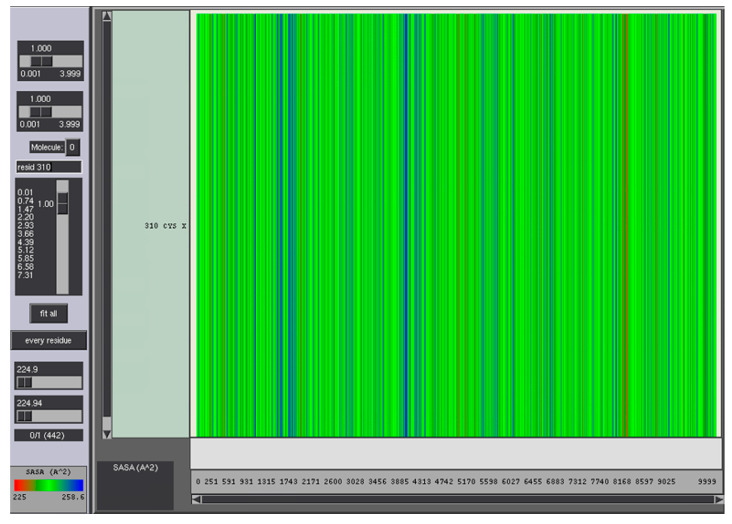
SASA analysis of cysteine residue in the linker.

A 100 ns MD simulation was performed to investigate the structural stability and binding affinity of scFv CR3022 toward RBD. Overall, the structure stability was evaluated by root-mean-square deviation, RMSD (Figure 3A). It was shown that the structure was relatively stable until the end of the simulation. The linear trendline of the RMSD is almost in a straight line with an average value of 2.8 Å. Furthermore, the residual fluctuation was calculated by the root-mean-square fluctuation, RMSF (Figure 3B). High fluctuations were observed in residues 143–150 and 309–324. These residues are located in the loop region and far from the epitope. Another high fluctuation was found in the linker of scFv, which consists of a loop structure. Moreover, the high occurrence of blue color (high solvent-accessible surface) in Figure 4 showed that the cysteine was mostly accessible to solvent, thus assuring the readiness to bind to the metal surface.

**Figure 5:**
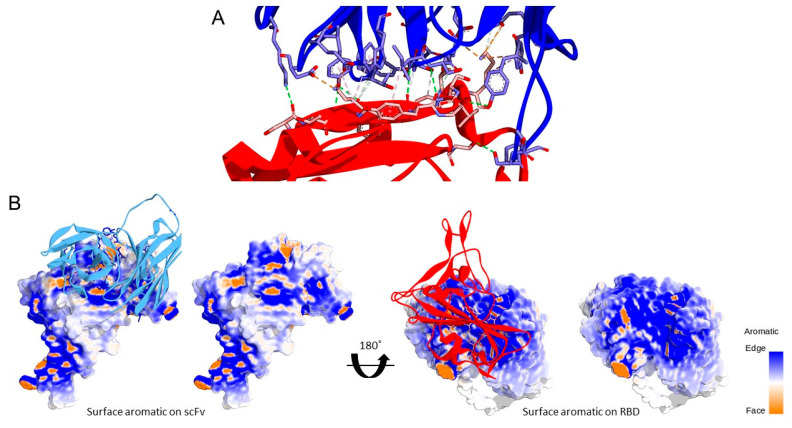
Molecular interaction between scFv in blue and RBD in red (**A**) Hydrogen bond is depicted with a green dashed line; the salt bridge is depicted with an orange dashed line, and hydrophobic interactions are depicted with a pink dashed line (**B**).

The molecular interaction between scFv CR3022-RBD of SARS-CoV-2 was analyzed to determine whether it still resembles the affinity of CR3022 Fab or not. The calculated MM/GBSA binding energy of scFv was −64.7 kcal/mol. These interactions are only composed of hydrophobic interactions and aromatic–aromatic interactions without calculating the entropy (Figure 5A). The scFv-RBD binding site is rich in aromatic residues that represent a typical antigen–antibody recognition pattern (Figure 5B) [22].

### 3.3. Production of scFv

**Figure 6:**
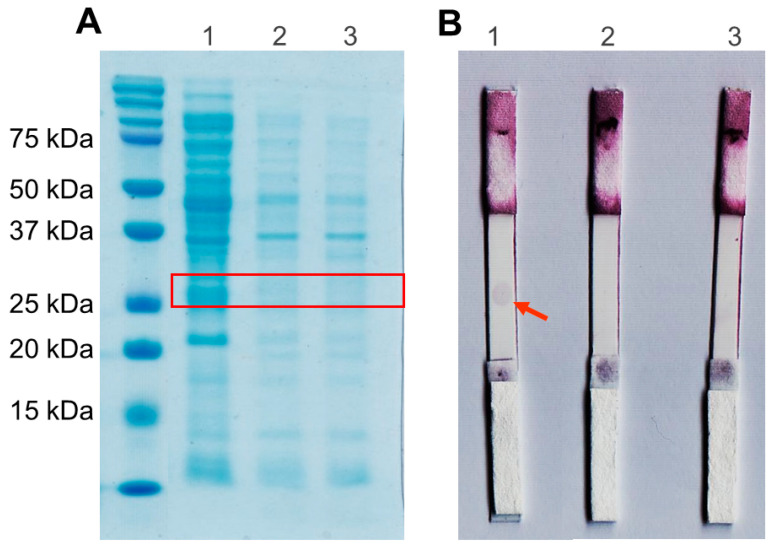
(**A**). SDS-PAGE electropherogram (1) the scFvs (2) periplasmic protein of *E. coli* BL21 (DE3) without recombinant plasmid IPTG induced (3) and IPTG uninduced (**B**). Spot test Analysis (1) 0.5 mg/mL scFv (2) periplasmic protein of *E. coli* BL21 (DE3) without recombinant plasmid (3) 0.5 mg/mL BSA in the third lane (**B**).

Electrophoregram SDS-PAGE showed that the scFv protein was successfully expressed in the periplasm, as indicated by the protein band above 25 kDa of the standard protein marker (Figure 6A). The theoretical molecular weight of scFv is 27 kDa, calculated by the Compute pI/Mw server (https://web.expasy.org/compute_pi/ (accessed on 1 December 2020). In addition, there was no protein band around 27 kDa in negative control lanes (*E. coli* BL21 (DE3) without recombinant plasmid IPTG induced and uninduced) (Figure 6A). This result indicates that the protein scFv specifically was expressed on *E. coli* BL21 (DE3)/pET-20b(+)-scFv.

### 3.4. Spot-Test Analysis

A positive result was determined when a spot appeared on nitrocellulose. In Figure 6B, the first lane shows that scFv produces a positive result. It indicates an interaction between scFv CR3022 and RBD SARS-CoV-2. As a negative control, the protein BSA and the periplasmic protein of *E. coli* BL21 (DE3) without recombinant plasmid is a crude protein with various proteins expressed natively. It shows that scFv has no cross-reactivity with the other protein.

### 3.5. Binding Kinetic Analysis Using Portable Surface Plasmon Resonance (SPR)

**Figure 7:**
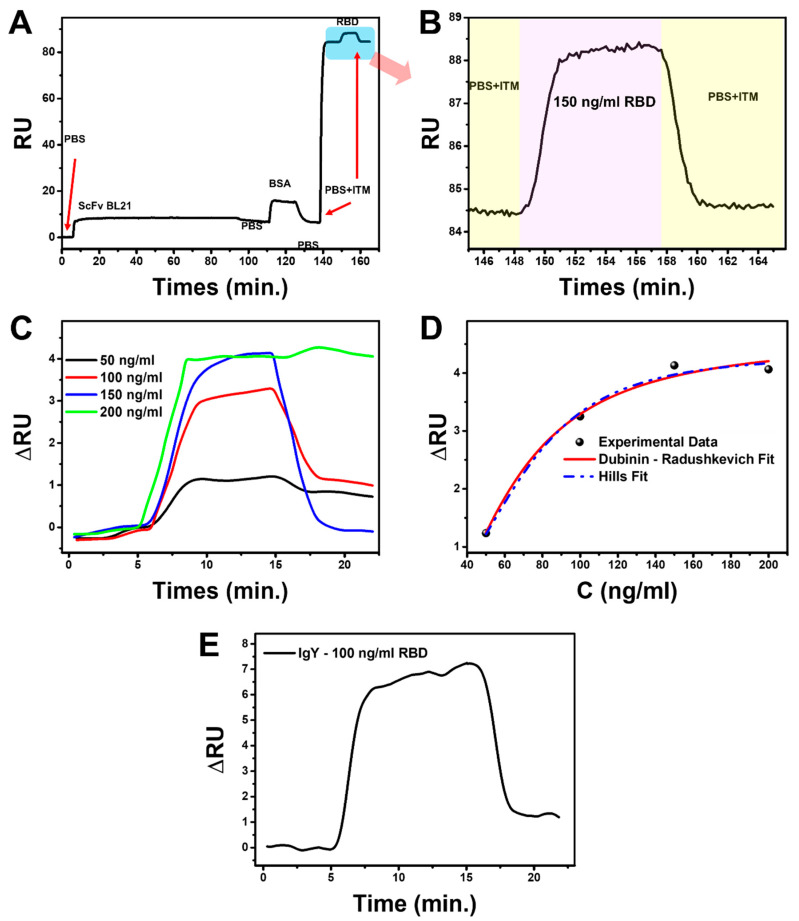
(**A**) Preparation of the SPR sensor chip: immobilization of scFv, blocking with BSA, and response signal scFv-RBD interactions, (**B**) magnification image of SPR sensorgram scFv-RBD binding interactions, (**C**) binding interaction between scFv and various concentrations of RBD, (**D**) Non-linear fitting of experimental data by Hills and Dubinin-Radushkevich adsorption isotherm model, and (**E**) SPR dynamic response of IgY to 100 ng/mL SARS-CoV-2 RBD.

In Figure 7A, during the immobilization of scFv, the sensorgram shows the SPR signal after the rinsing process does not return to the initial baseline; therefore, the immobilization process has been successful. Then, the blocking process using BSA shows that scFv has filled the SPR chip surface because rinsing with PBS causes the SPR signal to return to the baseline before the BSA injection process is carried out. PBS-ITM (Inactivation Transfer Medium) solution was used as the medium to represent the actual measurement conditions using the real swab sample for the RBD binding measurement process.

The results show the SARS-CoV-2 RBD binding process by scFv with a positive SPR signal (positive response) (Figure 7B). The measurement continued by varying the RBD concentrations to 50, 100, 150, and 200 ng/mL (Figure 7C). It was observed that the higher the RBD concentration, the higher the the SPR signal. The magnitudes of change in the Resonance Unit (RU) for 50, 100, 150, and 200 ng/mL concentrations were 1.13, 3.01, 3.74, and 3.98 RU, respectively.

The SPR data shows that the scFv can bind directly to RBD. The constant affinity was obtained using the non-linear regression method of the adsorption isotherm model in experimental data. In general, experimental data show that the adsorption characteristics of scFv-RBD follow the adsorption process of the Hills and Dubinin–Radushkevich (DR) model. We can obtain the affinity constant from the Hills isotherm adsorption model and predict the interaction mechanism. The equation is represented by Equation (1) [23].

(1) $$∆R=\left(\frac{∆R_{m}\cdot C_{\mathrm{eq}}{}^{\mathrm{nH}}}{K_{D}+C_{\mathrm{eq}}{}^{\mathrm{nH}}}\right)$$

∆R is the response when binding occurs, C is the SARS-CoV-2 RBD concentration, KD is the equilibrium dissociation constant, and nH is the Hills constant. In Figure 7D, the non-linear regression of the Hills isotherm model, with a relative coefficient (R^2^) of 0.9925, obtained KD of 11.54 nM and nH of 3.07. Based on the nH value, the scFv-RBD binding interaction occurs cooperatively positive because it has an nH > 1 [23,24]. It indicates that the RBD binding simplifies the other binding. Moreover, through the relationship of KD with Gibbs free binding energy (∆Gbind), the obtained ∆Gbind value is −10.82 kcal/mol.

Apart from the Hills model, the experimental data strongly correspond to the DR model. This model can obtain the adsorption mean free energy (E) and predict the adsorption process. The DR adsorption model is expressed by Equation (2) [25].

(2) $$∆R=∆R_{m}\;\exp\left\{-A_{D}{\left[\ln\left(1+\frac{1}{C_{\mathrm{eq}}}\right)\right]}^{2}\right\}$$

(3) $$A_{D}=B_{D}R^{2}T^{2}$$

(4) $$E=\frac{1}{\sqrt{2B_{D}}}$$

AD and BD are the constants related to the mean free energy of the adsorption SARS-CoV-2 RBD per mole by scFv, and this energy is expressed in Equation (4) [25,26]. R is the universal gas constant, and T is temperature. The DR model’s non-linear regression fitting in the experimental data has R^2^ of 0.9845 with the obtained parameter AD of 3309.69. The mean adsorption energy E ≈ 0.03 kJ/mol was calculated from this data by entering the R and T values at room temperature into Equations (3) and (4). In the DR isotherm model, if the E value is <8 kJ/mol, the adsorption process occurs in a physical nature [27]. Therefore, the positive cooperative interaction between Spike RBD of SARS-CoV-2 and scFv occurs by physisorption.

To compare the binding affinity of scFv to an antibody, we used an antibody (IgY) prepared based on our previous work [28]. The IgY concentration and experimental set used were the same as for scFv. However, the concentration was only 100 ng/mL, with the measurement results shown in Figure 7E. Using single-curve analysis in Anabel version 2.2.3 (anabel-online.com, BioCopy GmbH), the KD value obtained was 16.43 nM, and the ∆Gbind was −10,613 kcal/mol (the kinetic analysis curve and the resulting parameters can be seen in Figure S1 and Table S1, respectively, in Supplementary Materials). The affinity and binding energy between the scFv that we developed and IgY are similar.

### 3.6. Specificity Test

**Figure 8:**
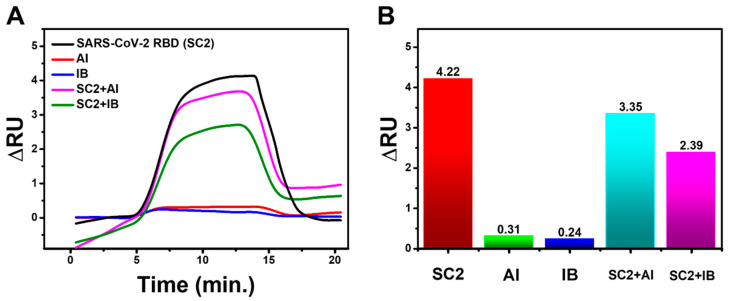
(**A**) SPR response dynamics and (**B**) bar chart of scFv response to AI, IB, and their mixture with Spike RBD of SARS-CoV-2 (SC2).

The developed scFv specificity was tested against avian influenza (AI), avian infectious bronchitis (IB) virus, and its mixture with Spike RBD SARS-CoV-2. Figure 8 compares the reactions, with the Spike RBD SARS-CoV-2 sample having a significantly higher response than the AI and IB viruses. The response to the mixed sample likewise exhibited relatively high results, demonstrating that the generated scFv is specific for the Spike RBD of SARS-CoV-2.

The specificity of scFv against SARS-CoV-2 RBD can be quantified using Equation (5) by considering the response values in the SARS-CoV-2 RBD samples, AI viruses, and IB viruses [29]. The response of each sample was 4.22 ru, 0.24 ru, and 0.31 ru, respectively, and from these values, a scFv specificity of 88.47% was obtained.

(5) $$Specificity=\left(\frac{true\;negatives\;responses}{true\;negatives\;responses+false\;positive\;responses}\right)\times 100\%$$

### 3.7. SARS-CoV-2 Virus Detection Test

Four nasopharyngeal samples from patients underwent the SARS-CoV-2 virus detection test, and the results were validated by PCR as positive or negative, as shown in the Table 1 below.

**Figure 9:**
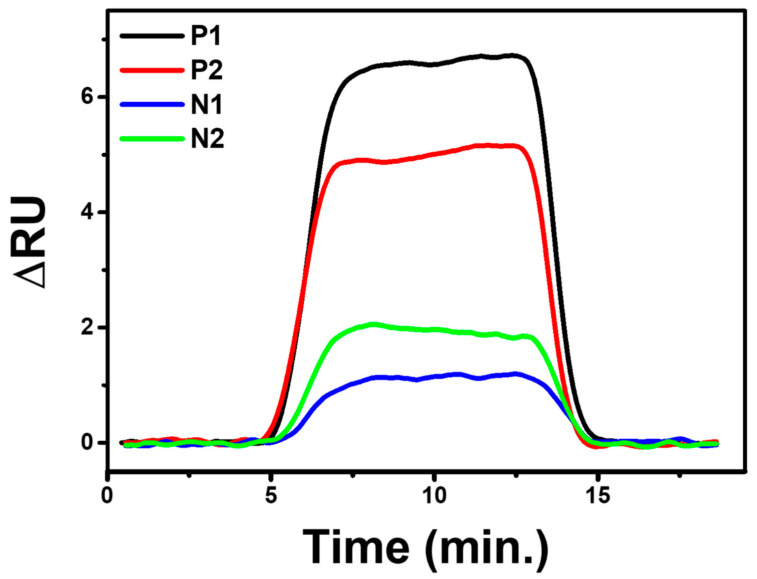
SPR response dynamics of the developed chip sensor to nasopharyngeal samples.

Figure 9 of the SPR dynamic response illustrates that positive samples have a higher ru value than negative samples. Positive 1 (P1), Positive 2 (P2), Negative 1 (N1), and Negative 2 (N2) samples’ dynamic response change values were 6.71 ru, 5.15 ru, 1.82 ru, and 1.17 ru, respectively. Unfortunately, negative samples have a 17–27% response to the highest ru values of positive samples. This irregularity may influence the viscosity and density of the flowing media, which changes as a result of the biological fluid of the nasopharyngeal swab sample. These two parameters can significantly affect the SPR measurement results because they can affect the adsorbed mass at the solid-liquid interface or the solvent content of the physisorbed layer [30,31]. Given that this preliminary study’s findings hint at scFv’s potential as a bioreceptor for the SARS-CoV-2 virus, it is worthwhile to continue researching it to employ it as a point-of-care diagnostic tool.

## 4. Discussion

This study demonstrated a straightforward method of developing scFv as a biosensor for plasmonic-based antigen detection for COVID-19. Recent studies have shown scFv as a ligand and have also reported on targeting applications in detecting viral infections [18,28]. Compared to the Fab format, scFv with a smaller molecular size has proper folding and assembly in the periplasm of *E. coli*. The Fab format also tends to form homo-dimers, which leads to decreased solubility and reduced expression level of Fab in *E. coli* [32,33]. RBD as target molecules in the spike (S) protein are abundantly expressed on virus surfaces. The S protein plays a critical role as a major antigen for virus entry by engaging the host receptor, mediating virus-host membrane fusion, and being the target of neutralizing antibodies [14,15]. Therefore, scFv may have a practical use in capturing antigenic particles of SARS-CoV-2.

In the structure of scFv, the linker serves to stabilize variable domains of light and heavy chains and increase affinity without altering the specificity. A linker that consists of 16 hydrophilic amino acids provides flexibility and solubility by preventing intercalation between peptides or variable domains during protein folding [34,35]. A metal-binding cysteine was also inserted into the linker to provide a free thiol group that allows site-directed coupling of the scFv with gold, or thiolated gold bond [36]. This ability was also supported by solvent-accessible surface area (SASA) analysis (Figure 4). Therefore scFv can be directly immobilized onto the gold surface in a favorable antigen-binding orientation at a high density which significantly increases the assay sensitivity by five-fold over the Fab antibody fragment [37].

The scFv is produced in the bacterial expression system of *E. coli* BL21 (DE3) to obtain a soluble and functional protein. This expression system is suitable for expressing small non-glycosylated recombinant antibody fragments [34,38]. In addition, *E. coli* strain BL21 (DE3) has a mutation in the ompT outer membrane protease that reduces proteolytic degradation in the extracellular space. For extracellular expression, the pelB signal peptide facilitated the transportation of the scFv into the *E. coli* periplasmic space with an oxidizing environment. It allowed the efficient formation of disulfide bonds as required by scFv [39,40]. To optimize expression conditions, we cultured *E. coli* BL21 (DE3)/pET-20b(+)-scFv at room temperature and low IPTG to maintain sufficiently low velocities, resulting in the facilitation of correct scFv folding into the soluble form [34,41]. Moreover, Terrific Broth (TB) was used as a rich medium that could increase protein yields greater than standard Luria Broth (LB) [42].

Soluble scFv was directly used as a bioreceptor without purification because scFv is designed to have a high affinity for the gold layer on the portable SPR chip, scFv(Sulfur)-Au Bond in Cysteine-Gold clusters. Gold is mostly used in SPR chips as it possesses stable chemical and optical properties; in comparison, silver has poor chemical stability and is highly susceptible to thermal desorption and oxidation, which precludes its wide use for SPR sensing. To increase the selectiveness between protein competitors and scFv toward the gold chip, we challenged it by flowing PBS solution at an optimal flow rate of 20 μL/min during the immobilization process (Figure 7A). PBS with a pH of 7.4 was selected as a running buffer because it is close to the theoretical pI of scFv (7.7). Generally, maximum adsorption of proteins occurs when the pH is close to their pI [43,44]. Our direct immobilization method has essential benefits such as speed, simplicity, favorable orientation, and increased functionality [45].

At 150 ng/mL, the SPR sensorgram dissociation curve showed a lower equilibrium response than the others (Figure 7C). This is due to the characteristics of the positively cooperative interaction in which the analyte–ligand binding will increase the affinity of the other analytes’ binding by the other ligands [46]. In this condition, a large concentration of analytes will increase the chances of aggregation because SARS-CoV-2 has hydrophobicity and thus can affect the analyte’s dissociation process [47,48]. Therefore, the difference in the dissociation curve pattern between the 150 ng/mL and 200 ng/mL concentrations and the lower concentrations occurs naturally to accommodate their different kinetic rates because the affinity of scFv at each concentration must be the same [49].

The antibody’s binding affinity to the antigen is essential in evaluating its potential for clinical use. Here, we calculated the MM/GBSA free energy of scFv to RBD of SARS-CoV-2 to be −64.7 kcal/mol. This favorable binding free energy indicates that the scFv derived from its Fab structure does not change its natural binding affinity towards the RBD antigen. In practical uses, the binding affinity of scFv to RBD SARS-CoV-2, determined by portable SPR spectroscopy, obtained a binding free energy of −10.822 kcal/mol. The binding free energy of scFv meets the high-affinity criteria because it is higher than −9 kcal/mol [50]. Nevertheless, improvement of the binding affinity of scFv can be addressed in future development.

## 5. Conclusions

In conclusion, the scFv from CR3022 antibody for Plasmonic-based Biosensor targeting the Spike RBD of SARS-CoV-2 has been successfully developed. MD simulations helped predict the binding stability of the scFv model with the target protein. Furthermore, MD simulations allowed us to determine the specific position of cysteine at the linker to make this scFv immobilize on a gold plate. The scFv was produced as a soluble protein in *E. coli* BL21 (DE3) and showed positive binding with the antigen protein in the spot test assay without cross-reactivity with the other protein, indicating its potential to be used in a lateral-flow-assay format as well. Moreover, the high binding affinity toward Spike RBD of SARS-CoV-2 was determined by portable SPR spectroscopy to be −10.82 kcal/mol. In addition to the antigenic protein, this scFv captured the SARS-CoV-2 virus from actual samples using SPR. We hope this study will help develop low-cost biosensors for future applications.

## Figures and Tables

**Table 1:** PCR data summary from nasopharyngeal swab samples.

No	Sample	PCR+/−	CT		
			RdRp	E	N
1	P1	+	33.95	27.25	31.59
2	P2	+	34.83	31.9	30.86
3	N1	−			
4	N2	−			

## Data Availability

Not applicable.

## Supplementary Materials

The following supporting information can be downloaded at: https://www.mdpi.com/article/10.3390/bios12121133/s1, Figure S1. Kinetic analysis of association (A) and dissociation (B) curves of IgY and Spike RBD SARS-CoV-2 100 ng/mL by Anabel version 2.2.3 software; Table S1. Obtained parameters of IgY kinetic analysis using the single-curve analysis method in Anabel 2.2.3.
