# Supplementary material for: Development of a Single-Chain Variable Fragment of CR3022 for a Plasmonic-Based Biosensor Targeting the SARS-CoV-2 Spike Protein
Source: Biosensors (Basel). 2022 Dec 6;12(12):1133. doi: 10.3390/bios12121133 (PMC9776105; doi:10.3390/bios12121133)
Supplement: Supplementary file 1 [file biosensors-12-01133-s001.zip › biosensors-1990100-supplementary.pdf]

Supplementary Materials

Development of a Single-Chain Variable Fragment of CR3022 for a Plasmonic-Based Biosensor Targeting the SARS-CoV-2 Spike Protein

Taufik Ramdani Tohari [1], Isa Anshori [2,3,*], Umi Baroroh [1,4], Antonius Eko Nugroho [2], Gilang Gumilar [3,5,6], Shinta Kusumawardani [1], Sari Syahruni [1], Brian Yuliarto [3,5], Wyanda Arnafia [7], Irvan Faizal [8,9], Yeni Wahyuni Hartati [1,10], Toto Subroto [1,10] and Muhammad Yusuf [1,10,*]

1. Research Center for Molecular Biotechnology and Bioinformatics, Universitas Padjadjaran, Bandung 40133, Indonesia
2. Lab-on-Chip Group, Biomedical Engineering Department, Institute of Technology, Bandung 40132, Indonesia
3. Research Center for Nanoscience and Nanotechnology (RCNN), Institut Teknologi Bandung, Bandung 40132, Indonesia
4. Department of Biotechnology, Indonesian School of Pharmacy, Bandung 40266, Indonesia
5. Advanced Functional Material Research Group, Faculty of Industrial Technology, Institut Teknologi Bandung, Bandung 40132, Indonesia
6. Research and Development Division, PT. Biostark Analitika Inovasi, Bandung 40375, Indonesia
7. Research and Development Division, PT. Tekad Mandiri Citra, Bandung 40292, Indonesia
8. Centre for Vaccine and Drug Research, National Research and Innovation Agency Republic of Indonesia, Kawasan Puspiptek Serpong, Tangerang Selatan 15314, Indonesia
9. Department of Biotechnology, Faculty of Biotechnology, Atma Jaya Catholic University of Indonesia, BSD Campus, Tangerang 15345, Indonesia
10. Department of Chemistry, Faculty of Mathematics and Natural Sciences, Universitas Padjadjaran, Jatinangor 45363, Indonesia

* Correspondence: isaa@staff.stei.itb.ac.id (I.A.); m.yusuf@unpad.ac.id (M.Y.)

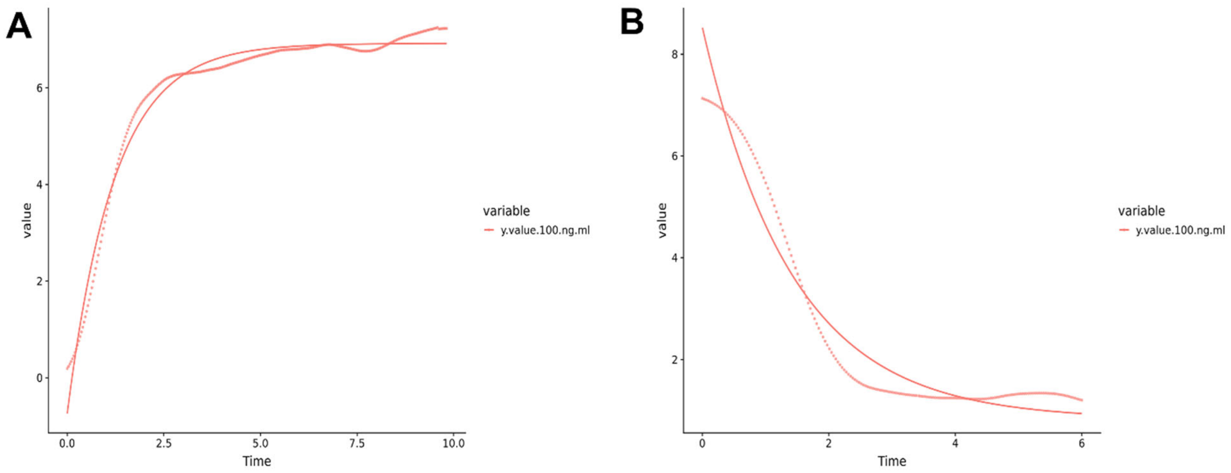

Figure S1. Kinetic analysis of association (A) and dissociation (B) curves of IgY-SARS-CoV-RBD 100 ng/ml by software anabel version 2.2.3

Table S1. Obtained parameters of IgY kinetic analysis using single curve analysis method in Anabel 2.2.3

Spot	c(Reagent) [M]	k_{obs}	k_{diss} [1/ min.]	k_{ass} [1/ min. M]	KD [M]
y.value.100.ng/ml	2.78E-09	0.822231	0.703297	42816122	1.64E-08

k_{obs}: Observation rate, k_{diss}: dissociation rate, k_{ass}: association rate, and KD: dissociation constant
